# Sarcasm and emoticons: Comprehension and emotional impact

**DOI:** 10.1080/17470218.2015.1106566

**Published:** 2015-12-11

**Authors:** Ruth Filik, Alexandra Țurcan, Dominic Thompson, Nicole Harvey, Harriet Davies, Amelia Turner

**Affiliations:** ^a^ School of Psychology, University of Nottingham, Nottingham, UK

**Keywords:** Language comprehension, Emotion, Figurative language, Sarcasm, Emoticons

## Abstract

Most theorists agree that sarcasm serves some communicative function that would not be achieved by speaking directly, such as eliciting a particular emotional response in the recipient. One debate concerns whether this kind of language serves to enhance or mute the positive or negative nature of a message. The role of textual devices commonly used to accompany written sarcastic remarks is also unclear. The current research uses a rating task to investigate the influence of textual devices (emoticons and punctuation marks) on the comprehension of, and emotional responses to, sarcastic versus literal criticism and praise, for both unambiguous (Experiment 1) and ambiguous (Experiment 2) materials. Results showed that sarcastic criticism was rated as less negative than literal criticism, and sarcastic praise was rated as less positive than literal praise, suggesting that sarcasm serves to mute the positive or negative nature of the message. In terms of textual devices, results showed that emoticons had a larger influence on both comprehension and emotional impact than punctuation marks.

Irony and sarcasm are forms of nonliteral language that are often used to communicate the opposite of what is literally said. Sarcasm is a specific form of irony, which is used when the target of the comment is a person (Kreuz & Glucksberg, 1989), and is the focus of this paper. Sarcastic comments are most commonly used to criticize someone (e.g., uttering, *You're early!* to a colleague who arrived late to a meeting), but they can also be used to praise (e.g., saying, *You're such a terrible tennis player!* to a friend who claims to not be able to play tennis, yet wins an important competition). Written sarcasm can be difficult to correctly understand due to the absence of the usual markers available in face-to-face conversations, such as tone of voice and facial expression. Thus, the use of sarcasm in a computer-mediated conversation can be risky, since the sender leaves open the possibility of the receiver interpreting the message literally. However, the use of emoticons and other devices could potentially aid in interpreting a sarcastic comment in this medium. To investigate this issue, we examine the comprehension and emotional impact of criticism and praise that are delivered either literally or sarcastically, and the influence of emoticons and punctuation marks on these aspects of communication.

## The emotional impact of sarcastic versus literal comments

Since sarcasm can be more difficult to process and understand than the same message intended literally (e.g., Filik, Leuthold, Wallington, & Page, 2014; Filik & Moxey, 2010; Kaakinen, Olkoniemi, Kinnari, & Hyönä, 2014; Regel, Gunter, & Friederici, 2011; Spotorno, Cheylus, Van Der Henst, & Noveck, 2013), there must be some benefit from using sarcasm in order to communicate, which may justify the increased risk of misunderstanding. In support of this, most researchers agree that the use of sarcasm serves some kind of social or emotional function (Brown & Levinson, 1987; Colston, 1997; Dews & Winner, 1995; Leech, 1983).

For example, according to the tinge hypothesis (e.g., Dews, Kaplan, & Winner, 1995; Dews & Winner, 1995), one function of sarcasm is to mute the emotional impact of both criticism and praise. In other words, sarcastic criticism will be perceived as less negative than literal criticism, while sarcastic praise will be perceived as less positive than literal praise. This is because the reader or hearer's perception of the intended meaning of a sarcastic comment will be “tinged” with the literal meaning. Specifically, sarcastic criticisms such as “That was clever”, will be tinged with the positive literal meaning of *clever*, and sarcastic praise such as “That was silly”, will be tinged with the negative literal meaning of *silly* (for evidence supporting this hypothesis, see Boylan & Katz, 2013; Harris & Pexman, 2003; Jorgensen, 1996; Matthews, Hancock, & Dunham, 2006).

In contrast, other researchers have found that the use of sarcasm actually enhances the emotional impact of the message (see e.g., Blasko & Kazmerski, 2006; Bowes & Katz, 2011; Colston, 1997; Filik, Hunter, & Leuthold, 2015; Kreuz, Long, & Church, 1991; Leggitt & Gibbs, 2000; Toplak & Katz, 2000). One potential explanation for this finding is that the speaker may choose to use sarcasm instead of speaking literally in order to additionally convey a negative attitude towards the recipient of the comment (Lee & Katz, 1998).

Since the existing evidence regarding the perceived emotional impact of sarcastic comments as compared to literal ones is mixed and conflicting, an initial aim of this paper is to examine whether the use of sarcasm enhances or mutes the positive or negative nature of criticism and praise.

## The role of emoticons in sarcasm comprehension

One of the key differences between computer-mediated communication (CMC, e.g., email, instant messaging, forum/blog posts, texting) and face-to-face communication (FTF) is that the former does not benefit from the nonverbal cues that are available in the latter, which are used to aid comprehension and remove ambiguities (Kiesler, Siegel, & McGuire, 1984). Psycholinguists have therefore been curious to investigate in what way, if any, users adapt their language in order to overcome these shortcomings of CMC. One of the main adaptations of language observed in CMC is the use of emoticons.

An emoticon (or emotional icon) is generally defined as “an ASCII glyph used to indicate an emotional state” (Wolf, 2000, p. 828). There now exists a very wide range of emoticons that are frequently used in CMC to express emotion, clarify the meaning of an ambiguous message, mark irony and sarcasm, or generally compensate for the lack of nonverbal cues (Derks, Bos, & von Grumbkow, 2008). Central to the current paper is the question of whether the use of emoticons (or other punctuation marks) can influence the comprehension and emotional impact of sarcastic messages in CMC.

Walther and D'Addario (2001) conducted one of the first studies to address this question. Their participants were asked to read emails including positive or negative messages, followed by a smiley face **:-)**, a sad face **:-(**, a wink face **;-)**, or no emoticon. Messages were ambiguous as to whether they were intended literally or sarcastically. Participants were asked to complete a questionnaire that contained questions relating to the writer's attitudes, feelings, intentions, ambiguity of the message, and the emotion transmitted by the message.

Results showed that participants associated the wink face with sarcasm in 85% of cases. Furthermore, the most sarcastic condition was a positive verbal message with a wink. However, this message–emoticon combination was not significantly more sarcastic than a positive message with a smile, a sad face, or nothing at all. Therefore, the authors concluded that winks do not actually connote greater sarcasm than other emoticons. In terms of valence, messages were not perceived differently when an emoticon was present versus absent, leading the authors to conclude that emoticons can complement a verbal message but cannot contradict or enhance its valence/meaning.

Derks et al. (2008) ran a similar study to Walther and D'Addario's (2001); examining the same set of emoticons, but included a neutral message condition (in addition to positive and negative), and the participants in the study were the recipients of the emails. In contrast to Walther and D'Addario (2001), Derks et al. showed that emoticons enhanced the valence of a message compared to a neutral valenced condition and compared to a positive or negative message without an emoticon. In terms of perceived sarcasm, they found that incongruent conditions (positive message with a negative emoticon or vice versa) lead to the perception of more sarcasm being expressed. Additionally, and again in contrast to Walther and D'Addario, messages with a wink face were rated as significantly more sarcastic than those without an emoticon.

Both studies mentioned above involved participants reading email messages that somebody else had written. In contrast to this, Hancock (2004) asked participants to chat with each other on given topics either via CMC or in FTF conditions. Analysis of these chats revealed that, although sarcasm was used more frequently in CMC than in FTF conditions, supportive cues were used less in CMC (where participants used cues such as ellipsis, punctuation, emoticons, etc.) than in FTF (where cues included prosody or laughter). Furthermore, punctuation marks (specifically, ellipsis, i.e., “ . . . ”) were more frequently used in CMC to mark sarcasm than emoticons. However, it should be noted that Hancock (2004) seems to also interpret incidental uses of ellipsis as marking sarcasm—for example, when ellipsis is simply used to mark a pause in a sarcastic sentence (see Vandergriff, 2013). Nonetheless, the sparse use of emoticons in Hancock's study seems to support Walther and D'Addario's (2001) conclusion that emoticons do not enhance the perception of a message as sarcastic.

Whalen, Pexman, and Gill (2009) conducted a similar study, in which participants were asked to write two emails to a friend, discussing past events and future plans, respectively. In this study, irony occurred in approximately 7.4% of cases and, when used, was 3.6 times more likely to be marked than unmarked. However, emoticons were rarely used, and, instead, exclamation marks were the most common device. These results appear to support Hancock's (2004) findings, but it is important to note that this result was across many different types of nonliteral language, not sarcasm specifically. It is also important to mention that in Whalen et al.’s (2009) experiment, “sarcastic” comments were almost always self-referential, rather than directed at the listener. In this case it is maybe not surprising that emoticons were not used, since presumably, emoticons would be used to help the receiver decode a sarcastic message that is addressed to them, because those comments carry a risk of being misinterpreted and may thus have a negative impact on the sender–receiver relationship. When sarcastic comments are self-referential, such a risk is not present.

From the studies outlined above, it is currently unclear whether emoticons such as the wink face either do (Derks et al., 2008; see also Attardo, Eisterhold, Hay, & Poggi, 2003) or do not (Walther & D'Addario, 2001) facilitate sarcastic language comprehension, or whether perhaps instead other punctuation devices are more useful (e.g., Hancock, 2004; Whalen et al., 2009). In the current paper, our aim is to systematically examine the influence of emoticons and other textual devices on the comprehension and emotional impact of different kinds of written sarcasm, under conditions in which the context either does or does not strongly support the sarcastic interpretation.

Thus, the present study examines the comprehension and emotional impact of sarcastic versus literal criticism and praise, and the influence of emoticons and other punctuations marks in CMC messages that are unambiguous (where the context clearly supports one interpretation; Experiment 1), or ambiguous (where the context does not support either the literal or the nonliteral interpretation; Experiment 2).

## EXPERIMENT 1

The aim of the first experiment was to investigate the comprehension and emotional impact of unambiguous CMC messages—that is, messages presented in contexts supporting either the literal or the sarcastic interpretation. Participants were presented with scenarios (see Table 1), in which the final comment should be interpreted literally or sarcastically—specifically, as sarcastic criticism, literal criticism, sarcastic praise, or literal praise. Comments could be accompanied by a wink emoticon ;-), tongue face emoticon ;-P, ellipsis . . . , exclamation mark !, or no punctuation. Participants were asked to rate the final comment of each scenario in terms of (a) how ironic they thought it was, and (b) how they thought the recipient of the comment would feel (from very negative to very positive).

**Table 1.** Example material in all experimental conditions in Experiment 1

Condition	
Sarcastic criticism (with device) Sarcastic criticism (no device) Literal criticism (with device) Literal criticism (no device) Sarcastic praise (with device) Sarcastic praise (no device) Literal praise (with device) Literal praise (no device)	Tanya had noticed that Jenny had put on a lot of weight. She texted her to say: “I see the diet is going well [:-P / ;-) /_ . . . _/!]” Tanya had noticed that Jenny had put on a lot of weight. She texted her to say: “I see the diet is going well” Tanya had noticed that Jenny had put on a lot of weight. She texted her to say: “I see the diet is going badly [:-P / ;-) /_ . . . _/!]” Tanya had noticed that Jenny had put on a lot of weight. She texted her to say: “I see the diet is going badly” Tanya had noticed that Jenny had lost a lot of weight. She texted her to say: “I see the diet is going badly [:-P / ;-) /_ . . . _/!]” Tanya had noticed that Jenny had lost a lot of weight. She texted her to say: “I see the diet is going badly” Tanya had noticed that Jenny had lost a lot of weight. She texted her to say: “I see the diet is going well [:-P / ;-) /_ . . . _/!]” Tanya had noticed that Jenny had lost a lot of weight. She texted her to say: “I see the diet is going well”

In terms of the effects of emoticons and punctuation, if it is the case that the wink emoticon is most effective as a sarcasm marker (e.g., Derks et al., 2008), then we would expect the wink emoticon to have the biggest influence on irony ratings. If, on the other hand, the ellipsis (e.g., Hancock, 2004) or exclamation mark (e.g., Whalen et al., 2009; see also Adams, 2012; Waseleski, 2006) is more effective, then we would expect the highest irony ratings in these conditions instead. For more exploratory purposes, we also tested the influence of the tongue face emoticon (following Carvalho, Sarmento, Silva, & de Oliveira, 2009; Garrison, Remley, Thomas, & Wierszewski, 2011). While this emoticon has not previously been individually examined as a marker of sarcasm, Carvalho et al. (2009) included it in a group of “positive emoticons” that they claimed are used to indicate sarcasm.

Following the tinge hypothesis (Dews & Winner, 1995), if the use of irony mutes the emotional impact of a message, then we would expect that messages intended as sarcastic criticism would be rated less negatively than messages that were intended as literal criticism. In addition, messages that were intended as sarcastic praise should be rated less positively than messages that were intended as literal praise. In contrast, if it is the case that sarcasm actually enhances the emotional impact of a message (e.g., Colston, 1997), we would expect to find the opposite (i.e., more extreme emotional ratings for sarcastic than for literal comments).

### Method

#### Participants

A total of 144 native English-speaking undergraduate and postgraduate students took part (113 females, *M*_age_ = 19 years and 7 months, *SD* = 1 year and 8 months).

#### Materials and design

Forty-eight scenarios were devised (see Table 1 for an example). Each item consisted of a context sentence followed by a description of one character sending a text message (or Facebook message) to the other character. The correct interpretation of the comment could be either literal or sarcastic and could be intended as either criticism or praise, depending on the context. The comment could be followed either by an emoticon/punctuation, or by no emoticon/punctuation, with the type of device being a between-subjects factor. Thus the experiment consisted of a 2 (literality: literal vs. sarcastic) × 2 (valence: criticism vs. praise) × 2 (device presence: device vs. no device) × 4 (device type: wink face ;-) vs. tongue face :-P vs. ellipsis vs. exclamation mark) mixed design. All factors were within items; literality, valence, and device presence were within subjects, and device type was between subjects. There were eight versions of the questionnaire such that each participant saw each item in only one of eight conditions (and saw only one type of device).

Each material was followed by questions relating to the comprehension of the comment (*Question a*), and to the emotional impact (*Question b*)^1^

^1^In both experiments, participants were also asked to rate how confident they were in their interpretation and how they thought the sender of the comment would have intended the recipient to feel. These data are not reported, since they do not add substantially to the manuscript, and are not published elsewhere.:

- *How ironic do you think the final comment is?*
  Not at all ironic 1 2 3 4 5 6 7 8 Very ironic
- *How do you think the recipient of the comment would feel?*
  Very negative 1 2 3 4 5 6 7 8 Very positive

#### Procedure

Participants were simply instructed to read the short scenarios and then answer the questions underneath by selecting a number on each scale. Once the task was completed, participants were allocated course credit for taking part and were debriefed.

### Results and discussion

Data analysis was performed in R (Version 3.0.2) using linear mixed modelling (lme4 package Version 1.0–5). The first step was to establish the appropriate random-effects structure for each analysis (that is, for each of the questions asked to the participants). The procedure followed that recommended by Barr, Levy, Scheepers, and Tily (2013). We started by fitting the full model to the data. The random-effects structure of the full model was: (1 + Literality × Valence × Device Presence|Subject) + (1 + Literality × Valence ×  Device Presence × Device Type|Item). The fixed-effects structure at this stage was Literality × Valence × Device Presence × Device Type, and it remained the same until the appropriate random-effects structure was found. The reason literality, valence, and device presence were introduced as random slopes for both subjects and items was that all factors were within subjects and within items, respectively. Device type was introduced as a random slope for items but not subjects, because it was a between-subjects and within-items factor. However, since the maximal model failed to converge (that is, the model could not be fitted to the data after 10,000 evaluations), the random-effects structure had to be simplified in order to obtain convergence. This was done by progressively removing one random slope at a time—the one that explained the least amount of variance in the previous nonconverging model.

Once the random-effects structure had been established, the second step was to perform a series of likelihood-ratio tests (chi-square tests) comparing the fit of models with different fixed-effects structures in order to reach the best model fit for our data. The procedure used was to compare the model with all four factors in interaction with progressively simpler fixed-effects structures (that is, removing one interaction at a time and then, when no more interactions remained, removing one main effect at a time). Table 2 shows the models that had the best fit for our data and the values of their fixed-effects parameters. The likelihood-ratio tests revealed that there was a significant four-way interaction. In order to decompose this interaction, we decided to run separate analyses for each device type.

**Table 2.** Best fitting models and fixed-effects parameters in Experiment 1

Question and device	Model	Fixed effects	Coefficient	*SE*	*t*
a ;-)	a ∼ Literality × Valence × Devicepresence + (1 + Literality|Subject) + (1 + Literality|Item)	(Intercept) literalityliteral valencepraise devicepresenceyes literalityliteral:valencepraise literalityliteral:devicepresenceyes valencepraise:devicepresenceyes literalityliteral:valencepraise:devicepresenceyes	7 −5.1 −0.3 −0.1 0.03 0.9 0.3 −0.7	0.2 0.2 0.1 0.1 0.2 0.2 0.2 0.2	39.5 −21.6 −2.8 −0.9 0.2 5.5 1.8 −3.1
a :-P	a ∼ Literality × Valence × Devicepresence + (1 + Literality|Subject) + (1 + Literality|Item)	(Intercept) literalityliteral valencepraise devicepresenceyes literalityliteral:valencepraise literalityliteral:devicepresenceyes valencepraise:devicepresenceyes literalityliteral:valencepraise:devicepresenceyes	6.8 −4.9 −0.5 −0.1 0.4 1.1 0.4 −1	0.2 0.3 0.1 0.1 0.2 0.2 0.2 0.3	34 −18.1 −3.8 −1.2 2 6.2 2 −3.7
a_ . . . _	a ∼ Literality × Devicepresence + (1 + Literality|Subject) + (1 + Literality|Item)	(Intercept) literalityliteral devicepresenceyes literalityliteral:devicepresenceyes	6.6 −4.6 0 0.2	0.2 0.2 0.1 0.1	38.1 −21.9 −0.1 1.9
a !	a ∼ Literality + Valence + (1 + Literality|Subject) + (1 + Literality|Item)	(Intercept) literalityliteral valencepraise	6.6 −4.7 −0.2	0.2 0.2 0.1	30.9 −19.6 −3.2
b ;-)	b ∼ Literality × Valence × Devicepresence + (1 + Valence|Subject) + (1 + Literality × Valence|Item)	(Intercept) literalityliteral valencepraise devicepresenceyes literalityliteral:valencepraise literalityliteral:devicepresenceyes valencepraise:devicepresenceyes literalityliteral:valencepraise:devicepresenceyes	3.3 −0.8 1.5 0.3 2.8 0.4 0.3 −1.2	0.1 0.1 0.2 0.1 0.3 0.1 0.1 0.2	24.9 −6.6 7.3 3.3 7.8 2.8 1.9 −5.7
b :-P	b ∼ Literality × Valence × Devicepresence + (1 + Valence|Subject) + (1 + Literality × Valence|Item)	(Intercept) literalityliteral valencepraise devicepresenceyes literalityliteral:valencepraise literalityliteral:devicepresenceyes valencepraise:devicepresenceyes literalityliteral:valencepraise:devicepresenceyes	3.2 −0.8 1.7 0.4 2.6 0.4 0.3 −1.4	0.2 0.1 0.2 0.1 0.2 0.1 0.1 0.2	15.4 −5.6 6.7 3.8 10.8 2.8 2.3 −6.2
b_ . . . _	b ∼ Literality × Valence + (1 + Valence|Subject) + (1 + Literality × Valence|Item)	(Intercept) literalityliteral valencepraise literalityliteral:valencepraise	3.1 −0.7 1.7 2.3	0.1 0.1 0.2 0.3	23.8 −5.7 6.7 8.1
b !	b ∼ Literality × Valence + Devicepresence + (1 + Valence|Subject) + (1 + Literality × Valence|Item)	(Intercept) literalityliteral valencepraise devicepresenceyes literalityliteral:valencepraise	3.3 −0.9 1.5 0.2 2.5	0.1 0.1 0.2 0.1 0.4	23.5 −6.2 7 3 5.6

#### Question a: How ironic do you think the final comment is?

##### Emoticons: Tongue face and wink

For the two emoticons, the literality of the comment interacted with valence and device presence, therefore the data were split into two categories—literal and sarcastic comments—and analyses were carried out separately for the two.

Literal comments were rated as more sarcastic when accompanied by an emoticon than without: *M*_criticism_with_:-P_ = 2.89, *SEM* = 0.13, *M*_criticism_without_:-P_ = 1.89, *SEM* = 0.1, χ^2^(1, *N* = 144) = 57.3, *p* < .001; *M*_criticism_with_;-)_ = 2.68, *SEM* = 0.13, *M*_criticism_without_;-)_ = 1.88, *SEM* = 0.09, χ^2^(1, *N* = 144) = 44.5, *p* < .001; *M*_praise_with_:-P_ = 2.15, *SEM* = 0.11, *M*_praise_without_:-P_ = 1.76, *SEM* = 0.08, χ^2^(1, *N* = 144) = 8.6, *p* < .01; *M*_praise_with_;-)_ = 1.95, *SEM* = 0.09; *M*_praise_without_;-)_ = 1.58, *SEM* = 0.07, χ^2^(1, *N* = 144) = 9.8, *p* < .01.

In contrast, sarcastic comments were not rated as more sarcastic when an emoticon was present versus absent: *M*_criticism_with_:-P_ = 6.65, *SEM* = 0.12, *M*_criticism_without_:-P_ = 6.8, *SEM* = 0.11, χ^2^(1, *N* = 144) = 1.37, *p* = .48; *M*_criticism_with_;-)_ = 6.91, *SEM* = 0.11, *M*_criticism_without_;-)_ = 7.02, *SEM* = 0.1, χ^2^(1, *N* = 144) = 0.9, *p* = .60; *M*_praise_with_:-P_ = 6.52, *SEM* = 0.13, *M*_praise_without_:-P_ = 6.3, *SEM* = 0.13, χ^2^(1, *N* = 144) = 2.76, *p* = .19; *M*_praise_with_;-)_ = 6.88, *SEM* = 0.1, *M*_praise_without_;-)_ = 6.69, *SEM* = 0.11, χ^2^(1, *N* = 144) = 2.8, *p* = .18.

These results suggest that both the wink emoticon and tongue face emoticon may mark sarcasm, but they seem to only increase the perceived sarcasm of literal comments, not of comments that are already sarcastic.

##### Punctuation: Ellipsis and exclamation mark.

The presence of ellipsis increased the perceived sarcasm of literal comments, *M*_literal_with_ . . . _= 2.22, *SEM* = 0.07, *M*_literal_without_ . . . _= 2.01, *SEM* = 0.06, χ^2^(1, *N* = 144) = 6.75, *p* = .01, but did not increase the perceived sarcasm of sarcastic comments, *M*_sarcastic_with_ . . . _= 6.63, *SEM* = 0.07, *M*_sarcastic_without_ . . . _= 6.64, *SEM* = 0.07, χ^2^(1, *N* = 144) = 0.007, *p* = 1. The presence of an exclamation mark did not have any effect on the perceived sarcasm of either literal or sarcastic comments.

##### Which device produces the greatest increase in perceived sarcasm?

**Figure 1:**
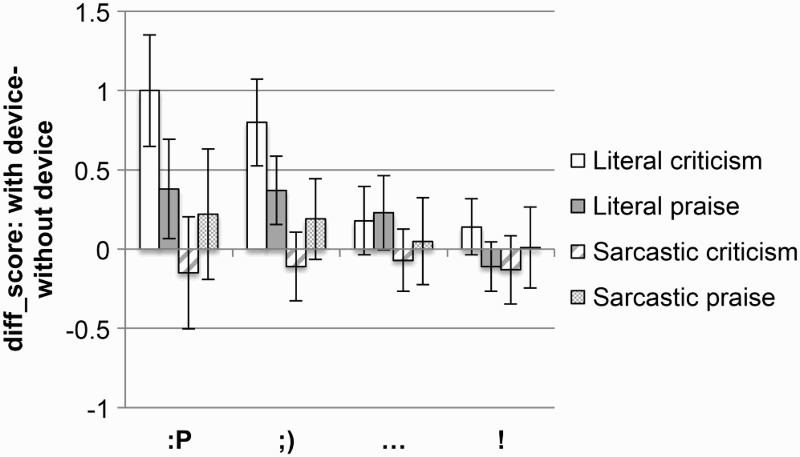
Difference sarcasm rating scores for literal and sarcastic praise and criticism, for each device. Error bars represent 95% CI (confidence interval).

In order to answer this question, a difference score was calculated for each device, and for each valence and literality, by subtracting the rating when the comment was not accompanied by the device from the rating when the comment was accompanied by the device (see Figure 1). Since we were specifically interested in comparing between the magnitudes of the effects of the different devices, targeted comparisons were conducted in the form of *t* tests.

Results indicated that the two emoticons (tongue face and wink) do not differ from each other in the amount by which they increase the perceived sarcasm of a comment, be it literal criticism, *t*(90) = 0.8, *p* = .4, literal praise, *t*(83) = 0.03, *p* = .9, sarcastic criticism, *t*(79) = −0.2, *p* = .8, or sarcastic praise, *t*(80) = 0.1, *p* = .9.

However, both emoticons had a significantly bigger effect on the perceived sarcasm of literal criticism than either of the punctuation marks: tongue face versus ellipsis, *t*(79) = 3.8, *p* < .001, wink versus ellipsis, *t*(88) = 3.2, *p* = .001, tongue face versus exclamation mark, *t*(72) = 4.1, *p* < .001, wink versus exclamation mark, *t*(81) = 3.7, *p* < .001. Furthermore, the two emoticons and ellipsis all had a similar effect on literal praise: tongue face versus ellipsis, *t*(85) = 0.7, *p* = .4, wink versus ellipsis, *t*(93) = 0.8, *p* = .4, increasing its perceived sarcasm significantly more than an exclamation mark did: tongue face versus exclamation mark, *t*(70) = 2.6, *p* = .01, wink versus exclamation, *t*(87) = 3.3, *p* = .001, ellipsis versus exclamation mark, *t*(85) = 2.3, *p* = .02. The two punctuation marks also had similar effects on sarcastic comments (all *t*s < 1).

#### Question b: How do you think the recipient of the comment would feel?

##### Emoticons: Tongue face and wink.

Both emoticons had identical effects on perceived emotional impact. For literal comments, the presence of an emoticon made criticism appear less negative: *M*_with_:-P_ = 3.27, *SEM* = 0.08, *M*_without_:-P_ = 2.4, *SEM* = 0.08, χ^2^(1, *N* = 144) = 78.2, *p* < .001; *M*_with_;-)_ = 3.25, *SEM* = 0.08, *M*_without_;-)_ = 2.48, *SEM* = 0.07, χ^2^(1, *N* = 144) = 71, *p* < .001. However, they had no effect on literal praise: *M*_with_:-P_ = 6.64, *SEM* = 0.08, *M*_without_:-P_ = 6.79, *SEM* = 0.08, χ^2^(1, *N* = 144) = 2.1, *p* = .3; *M*_with_;-)_ = 6.62, *SEM* = 0.08, *M*_without_;-)_ = 6.76, *SEM* = 0.08, χ^2^(1, *N* = 144) = 2.2, *p* = .28.

For sarcastic comments, an emoticon made criticism appear less negative, and praise appear more positive: *M*_criticism_with_:-P_ = 3.6, *SEM* = 0.09, *M*_criticism_without_:-P_ = 3.18, *SEM* = 0.09, χ^2^(1, *N* = 144) = 13.3, *p* < .001; *M*_criticism_with_;-)_ = 3.66, *SEM* = 0.09, *M*_criticism_without_;-)_ = 3.31, *SEM* = 0.09, χ^2^(1, *N* = 144) = 10.3, *p* = .002; *M*_praise_with_:-P_ = 5.7, *SEM* = 0.1, *M*_praise_without_:-P_ = 4.92, *SEM* = 0.11, χ^2^(1, *N* = 144) = 45.6, *p* < .001; *M*_praise_with_;-)_ = 5.45, *SEM* = 0.1, *M*_praise_without_;-)_ = 4.81, *SEM* = 0.11, χ^2^(1, *N* = 144) = 34.7, *p* < .001.

##### Punctuation marks: Ellipsis and exclamation mark

The presence of an exclamation mark makes comments more positive, irrespective of their literality or valence: *M*_with !_ = 4.5, *SEM* = 0.07, *M*_without !_ = 4.3, *SEM* = 0.07. However, the presence of ellipsis did not affect the perceived emotional impact of any of the comments.

##### Which device produces the biggest increase in perceived emotional impact?

**Figure 2:**
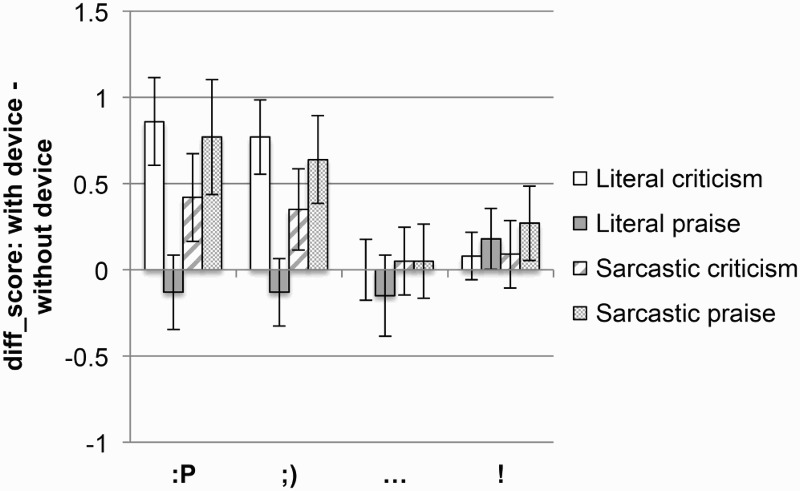
Difference emotional rating scores for literal and sarcastic praise and criticism, for each device. Error bars represent 95% CI (confidence interval).

To answer this question, a difference score was calculated in a similar way to that described under *Question a* (see Figure 2).

Results indicate that the two emoticons do not differ from each other in the amount by which they increase the perceived positivity of a comment, be it literal or sarcastic, criticism or praise (all *t*s < 1).

However, both emoticons had a significantly bigger effect on the perceived positivity of literal criticism and sarcastic praise than either of the punctuation marks, with no difference between the punctuation marks [literal criticism: tongue face vs. ellipsis, *t*(82) = 5.3, *p* < .001, wink vs. ellipsis, *t*(88) = 5.2, *p* < .001, tongue face vs. exclamation mark, *t*(74) = 5.0, *p* < .001, wink vs. exclamation mark, *t*(79) = 4.9, *p* < .001, ellipsis vs. exclamation mark, *t*(91) = −0.7, *p* = .4; sarcastic praise: exclamation mark vs. tongue face, *t*(80) = 2.3, *p* = .02, exclamation vs. wink, *t*(91) = 2.0, *p* = .03, ellipsis vs. tongue face, *t*(82) = 3.3, *p* = .001, ellipsis vs. wink, *t*(92) = 3.2, *p* = .001, ellipsis vs. exclamation mark, *t*(93) = −1.3, *p* = .1].

Furthermore, the two emoticons and ellipsis all made literal praise seem less positive, whereas the exclamation mark made it seem more positive: tongue face versus ellipsis, *t*(93) = 0.1, *p* = .9, wink versus ellipsis, *t*(92) = 0.1, *p* = .8, tongue face versus exclamation mark, *t*(90) = −2.2, *p* = .02, wink versus exclamation mark, *t*(91) = −2.2, *p* = .02, ellipsis versus exclamation mark, *t*(87) = −2.2, *p* = .02.

For sarcastic criticism, the exclamation mark had a similar effect to the wink and ellipsis and only had a marginally smaller effect than the tongue face [exclamation mark vs. wink, *t*(91) = 1.6, *p* = .1, exclamation mark vs. ellipsis, *t*(93) = −0.2, *p* = .7, exclamation vs. tongue face, *t*(87) = 1.9, *p* = .05], while the ellipsis had a smaller effect than both emoticons [ellipsis vs. tongue face, *t*(86) = 2.2, *p* = .02, ellipsis vs. wink, *t*(90) = 1.8, *p* = .06].

In conclusion, it seems that ellipsis affected the perceived sarcasm of literal but not of sarcastic comments, but did not influence the perceived emotional impact. Conversely, the exclamation mark did not affect the sarcasm ratings of any comments, but seemed to make all comments more positive.

#### The tinge hypothesis

**Figure 3:**
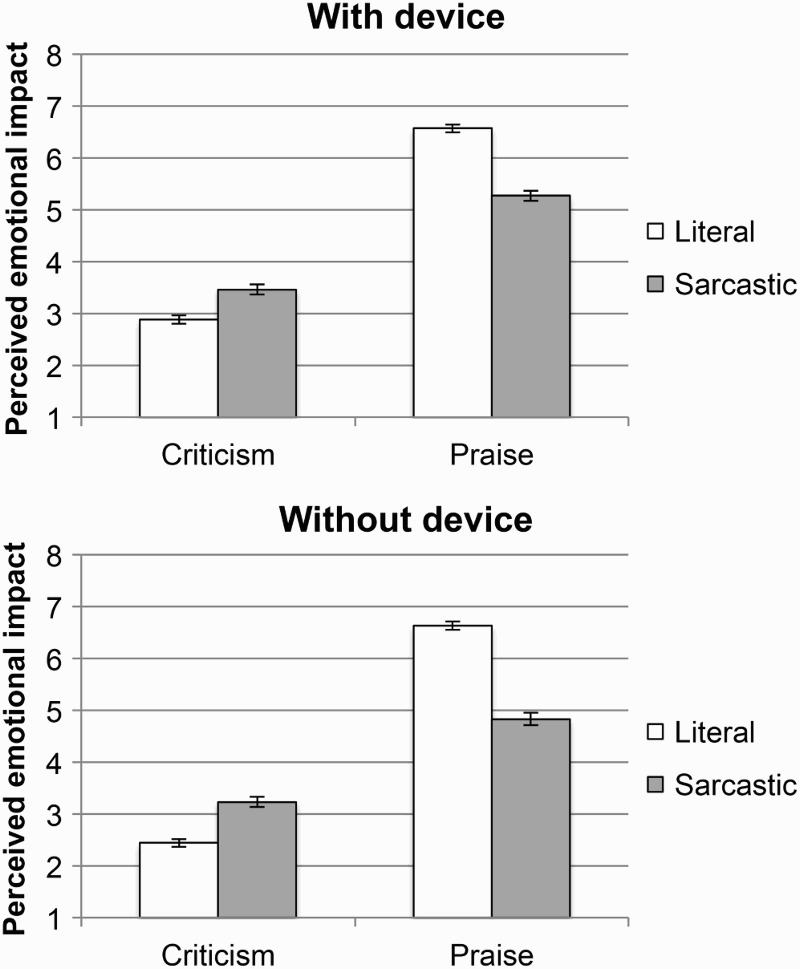
Mean perceived emotional impact of literal and sarcastic comments of both valences, in the presence of a device (top panel), and in the absence of a device (bottom panel). Error bars represent 95% CI (confidence interval).

The tinge hypothesis makes two predictions: that sarcastic criticism would be perceived as less negative than literal criticism, and sarcastic praise would be perceived as less positive than literal praise. When the comments were accompanied by a device, sarcastic criticism was perceived as less negative than literal criticism, χ^2^(1, *N* = 144) = 6.2, *p* = .02, and sarcastic praise was perceived as less positive than literal praise, χ^2^(1, *N* = 144) = 18.2, *p* < .001. The same pattern of results was observed when the comments were not accompanied by any device (see Figure 3): Sarcastic criticism was perceived as less negative than literal criticism, χ^2^(1, *N* = 144) = 7.9, *p* = .009, and sarcastic praise was perceived as less positive than literal praise, χ^2^(1, *N* = 144) = 19.3, *p* < .001.

These results fully support the tinge hypothesis. The only minor exception was for comments accompanied by a tongue face, where literal and sarcastic criticism did not significantly differ from each other, although descriptively the results were in the expected direction (with sarcastic criticism appearing less negative than literal criticism).

## EXPERIMENT 2

Results from Experiment 1 showed that textual devices had little impact on the comprehension of a sarcastic comment when the sarcastic interpretation was facilitated by the context. Thus, it is of interest to investigate the influence of textual devices on the perception of sarcasm when there is no such support available from the context. Therefore, the aim of the second experiment was to investigate the comprehension and emotional impact of ambiguous CMC messages.

In addition to the difference in the level of contextual support, the design of Experiment 2 was modified in a number of respects compared to Experiment 1. First, we wanted to manipulate “device” as a within-subjects variable, which necessitated reducing the number of devices investigated. Thus we decided to drop the tongue face emoticon, since the inclusion of this device in Experiment 1 was largely exploratory, and the results showed that it had almost identical effects to the wink emoticon. We also dropped the exclamation mark, since its effects did not seem to be specifically related to sarcasm.

Furthermore, since the materials in the current experiment were designed to be ambiguous, we asked participants to judge them on a 7-point scale, rather than the 8-point scale employed in Experiment 1, so that they could use the midpoint on the scale to indicate ambiguity. Finally, since all of the materials employed in both experiments involved a specific type of irony (i.e., sarcasm, which we simply define as an ironic comment that is aimed at a person), we asked participants to rate materials in terms of how sarcastic they were, instead of how ironic they were.

Participants were presented with scenarios (see Table 3), in which the final comment was ambiguous in terms of whether it should be interpreted literally or sarcastically. Comments could have either a positive valence (e.g., *I thought it was so interesting*), which could be interpreted as either literal praise or sarcastic criticism, or a negative valence (e.g., *I thought it was so boring*), which could be interpreted as either literal criticism or sarcastic praise. Comments could be accompanied by a wink emoticon **;-)**, ellipsis . . . , or simply a full stop. Participants were asked to rate the final comment of each scenario in terms of (a) how sarcastic they thought the speaker of the comment was being, and (b) how they thought the recipient of the comment would respond emotionally (from very positively to very negatively).

**Table 3.** Example material in all experimental conditions in Experiment 2

Device	Positive valence	Negative valence
Full stop	Person A: How did you find your presentation went earlier? Person B: I have no idea really. Person A: I thought it was so interesting.	Person A: How did you find your presentation went earlier? Person B: I have no idea really. Person A: I thought it was so boring.
Wink emoticon	Person A: How did you find your presentation went earlier? Person B: I have no idea really. Person A: I thought it was so interesting ;-)	Person A: How did you find your presentation went earlier? Person B: I have no idea really. Person A: I thought it was so boring ;-)
Ellipsis	Person A: How did you find your presentation went earlier? Person B: I have no idea really. Person A: I thought it was so interesting_ . . . _	Person A: How did you find your presentation went earlier? Person B: I have no idea really. Person A: I thought it was so boring_ . . . _

In terms of the effects of emoticons and punctuation, if it is the case that the wink emoticon is most effective as a sarcasm marker (e.g., Derks et al., 2008), then we would expect higher sarcasm ratings for comments accompanied by a wink than for those accompanied by ellipsis or a full stop. If, on the other hand, the ellipsis is more effective (e.g., Hancock, 2004), then we would expect the highest sarcasm ratings in this condition instead. We also aimed to assess whether our finding from Experiment 1—that sarcasm mutes the positive or negative nature of a message—would replicate in cases where the sarcasm was more ambiguous.

### Method

#### Participants

A new sample of 48 native English-speaking undergraduate students (34 females, *M*_age_ = 20 years and 6 months, *SD* = 10 months) took part.

#### Materials and design

Thirty-six scenarios were created (see Table 3 for an example). Each scenario depicted a short conversation that consisted of three messages between two people: Person A and Person B. While the topic of each conversation varied, all followed the same format with Person A messaging first, Person B replying, and Person A responding with the final comment. The final comment was “targeted” towards Person B, and the context was ambiguous as to whether it was intended to be sarcastic or not. Each of these 36 conversations was then split into two different versions, with the final comment altered slightly. One of the versions ended with a superficially positive comment (e.g., *I thought it was so interesting*), and one ended with a superficially negative comment (e.g., *I thought it was so boring*). Both the positive and negative comments then ended with a full stop, a wink emoticon, or ellipsis. This gave three positive valence messages and three negative valence messages for each scenario, resulting in six different versions for each of the 36 scenarios. Thus the experiment consisted of a 2 (valence: positive vs. negative) × 3 (device: full stop vs. wink emoticon vs. ellipsis) within-subjects design. Additionally, both factors were also within items, since each scenario had a version that ended with each of the three devices, and also each scenario had a positive and a negative target utterance. The experimental materials were counterbalanced by creating six versions of the questionnaire such that each participant saw each item in only one of the six conditions.

Each of the materials was followed by questions relating to the comprehension of the comment (*Question a*), and the emotional impact (*Question b*; see Footnote 1):

- *How sarcastic do you think Person A is being?*
  Not at all 1 2 3 4 5 6 7 Very
- *How do you think Person B will respond emotionally?*
  Very negatively 1 2 3 4 5 6 7 Very positively

#### Procedure

Participants were asked to read the scenarios and to indicate their response to each of the questions by highlighting the appropriate number on the scale. After the participants had completed the questionnaire, they were debriefed by the researcher and allocated course credit for taking part.

### Results and discussion

As in Experiment 1, data were analysed in R using linear mixed modelling. We started by fitting the full model to the data. The random-effects structure of the full model was: (1 + Valence × Device|Subject) + (1 + Valence × Device|Item). The fixed-effects structure at this stage was Valence × Device, and it remained the same until the appropriate random-effects structure was found. The reason valence and device were introduced as random slopes both for subjects and for items is because both factors were within subjects and within items, respectively. From here onwards, the analysis followed the same steps as those described in Experiment 1. Specifically, the procedure used was to compare the model with the two factors in interaction with progressively simpler fixed-effects structures (that is, two main effects but no interaction, or only one main effect). Table 4 shows the models that had the best fit for our data and the values of their fixed-effects parameters.

**Table 4.** Best fitting models and fixed-effects parameters in Experiment 2

Question	Model	Fixed effects	Coefficient	*SE*	*t*
a	a ∼ Valence × Device + (1 + Valence|Subject) + (1|Item)	(Intercept) valencepositive devicefullstop devicewinkface valencepositive:devicefullstop valencepositive:devicewinkface	3.6 0.5 −0.6 2.1 −1.6 −1.24	0.1 0.2 0.1 0.1 0.2 0.2	25.9 3.4 −4.9 15.2 −8.3 −6.3
b: sarcastic interpretation	b ∼ Valence × Device + (1 + Valence|Subject) + (1|Item)	(Intercept) valencepositive devicefullstop devicewinkface valencepositive:devicefullstop valencepositive:devicewinkface	3.8 −0.4 −0.2 1 0.6 −0.8	0.1 0.2 0.1 0.1 0.3 0.1	24.9 −2 −1.2 8.8 2.1 −5.6
b: literal interpretation	b ∼ Valence × Device + (1 + Valence|Subject) + (1|Item)	(Intercept) valencepositive devicefullstop devicewinkface valencepositive:devicefullstop valencepositive:devicewinkface	2.2 3.1 −0.1 0.9 0.5 −0.9	0.1 0.1 0.1 0.2 0.1 0.2	23.7 21.3 −1.6 5.5 3.7 −4.1

#### Question a: How sarcastic do you think Person A is being?

**Figure 4:**
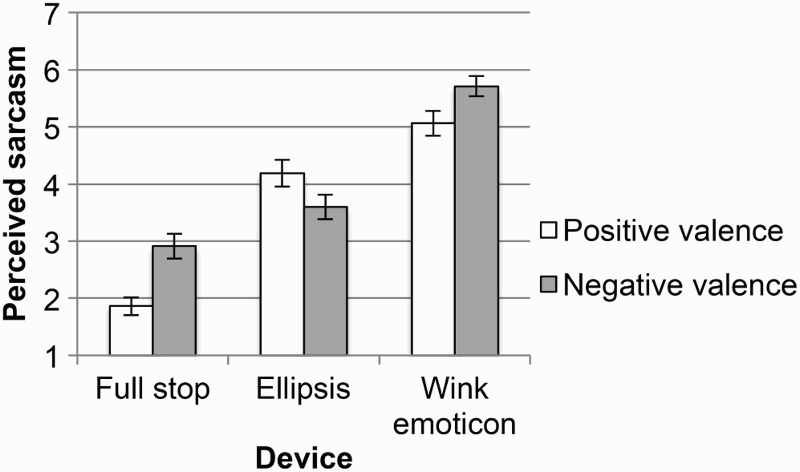
Mean sarcasm ratings. Error bars represent 95% CI (confidence interval).

There was an interaction between the valence of the comment and the type of device that accompanied the comment (see Figure 4). Positive comments were rated as most sarcastic when accompanied by a wink emoticon, less sarcastic when accompanied by ellipsis, and least sarcastic when accompanied by a full stop, χ^2^(2, *N* = 48) = 562.9, *p* < .001. Negative comments followed the same pattern, χ^2^(2, *N* = 48) = 433.3, *p* < .001 (all reported *p*-values are Bonferroni adjusted).

These results indicate that the wink emoticon promoted a sarcastic interpretation more strongly than ellipsis or a full stop for both positive and negative comments. This is in line with the results of Derks et al. (2008), who concluded that the wink emoticon makes ambiguous comments seem more sarcastic; however, it contradicts the findings of Walther and D'Addario (2001), who concluded that the wink emoticon does not significantly increase the interpretation of ambiguous messages as sarcastic. The findings of Hancock (2004) are also not fully supported, since ellipsis was not the device most associated with sarcasm.

#### Question b: How do you think Person B will respond emotionally?

We were interested in how the different emoticons affected the emotional perception of a comment depending on how that comment was interpreted. Therefore, the data were divided into comments that had received a sarcastic interpretation and comments that had received a literal interpretation, and separate analyses were performed in order to answer the specific research question outlined above. Specifically, comments of positive valence with a sarcasm rating of more than 4 (on the scale from 1 to 7) were considered sarcastic criticism, while comments of negative valence with a sarcasm rating of more than 4 were considered sarcastic praise. Comments of positive valence with a sarcasm rating of less than 4 were considered literal praise, while comments of negative valence with a sarcasm rating of less than 4 were considered literal criticism. Comments with sarcasm ratings of exactly 4 (19.5% of the data) were removed from the analysis.

**Figure 5:**
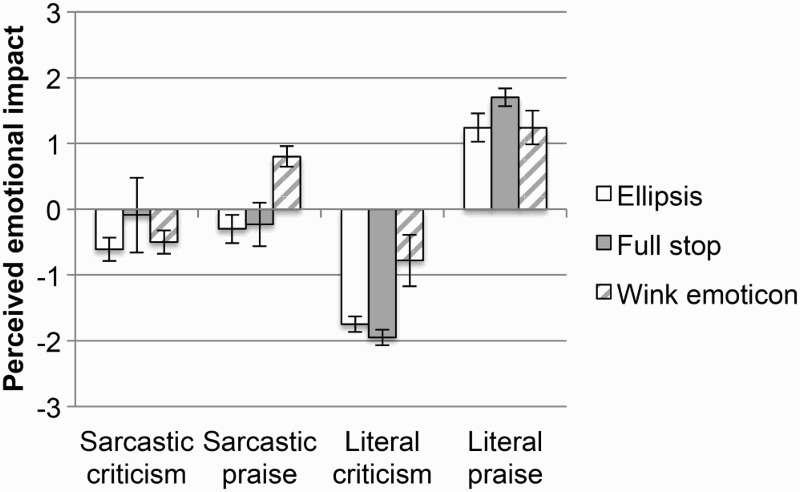
Mean perceived emotional impact. The rating scale has been converted so that 0 represents the middle rating of 4, while positive scale numbers represent ratings from 5 to 7, and negative numbers represent ratings from 1 to 3. Error bars represent 95% CI (confidence interval).

*Sarcastic criticism* had a less negative emotional impact when accompanied by a full stop than when accompanied by ellipsis, while comments accompanied by a wink did not differ from the other two conditions (see Figure 5): full stop versus ellipsis, χ^2^(1, *N* = 48) = 8.86, *p* = .005; full stop versus wink, χ^2^(1, *N* = 48) = 0.06, *p* = 1; wink versus ellipsis, χ^2^(1, *N* = 48) = 0.38, *p* = .38.

*Sarcastic praise* had a more positive emotional impact when accompanied by a wink than when accompanied by either ellipsis or a full stop, with no difference between the latter two devices (see Figure 5): wink versus ellipsis, χ^2^(1, *N* = 48) = 77.5, *p* < .001, wink versus full stop, *χ* ^2^ (1, *N* = 48) = 70.1, *p* < .001, full stop versus ellipsis, χ^2^(1, *N* = 48) = 0.47, *p* = .98.

*Literal criticism* was perceived as less negative when accompanied by a wink than when accompanied by ellipsis or a full stop, with no difference between the latter two devices (see Figure 5): wink versus ellipsis, χ^2^(1, *N* = 48) = 31.4, *p* < .001, wink versus full stop, χ^2^(1, *N* = 48) = 41.5, *p* < .001, full stop versus ellipsis, χ^2^(1, *N* = 48) = 3.3, *p* = .13.

*Literal praise* was perceived as more positive when accompanied by a full stop than when accompanied by a wink or ellipsis, with no difference between the latter two devices (see Figure 5): full stop versus ellipsis, χ^2^(1, *N* = 48) = 13.2, *p* < .001, full stop versus wink, χ^2^(1, *N* = 48) = 7.7, *p* = .01, wink versus ellipsis, χ^2^(1, *N* = 48) = 0.002, *p* = 1. For a summary of the results of Experiments 1 and 2, please refer to Table 5.

**Table 5.** Overview of results from Experiments 1 and 2

		Sarcasm rating	Positivity of emotional rating
Experiment 1	Sarcastic criticism	with :-P = without :-P with ;-) = without ;-) with . . . = without … with ! = without !	with :-P > without :-P with ;-) > without ;-) with . . . = without … with ! > without !
	Literal praise	with :-P > without :-P with ;-) > without ;-) with . . . > without … with ! = without !	with :-P = without :-P with ;-) = without ;-) with . . . = without … with ! > without !
	Sarcastic praise	with :-P = without :-P with ;-) = without ;-) with . . . = without … with ! = without !	with :-P > without :-P with ;-) > without ;-) with . . . = without … with ! > without !
	Literal criticism	with :-P > without :-P with ;-) > without ;-) with . . . > without … with ! = without !	with :-P > without :-P with ;-) > without ;-) with . . . = without … with ! > without !
Experiment 2	Sarcastic criticism	;-) > . . . > full stop	full stop > [ … = ;-)]
	Literal praise		full stop > [ … = ;-)]
	Sarcastic praise	;-) > . . . > full stop	;-) > (full stop = … )
	Literal criticism		;-) > (full stop = … )

These findings partially support the results of Derks et al. (2008) and contradict those of Walther and D'Addario (2001), by showing that the use of a wink emoticon can make superficially negative comments (i.e., sarcastic praise and literal criticism) seem less negative. It does not, however, make superficially positive comments (i.e., sarcastic criticism and literal praise) seem more positive.

#### The tinge hypothesis

**Figure 6:**
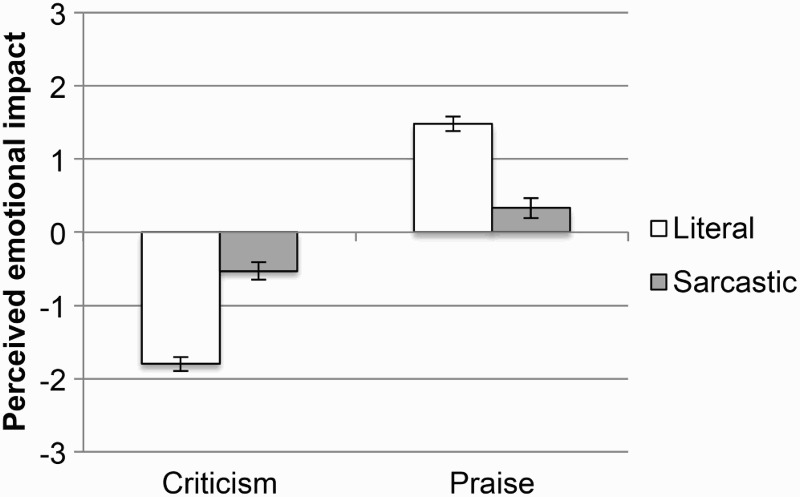
Mean perceived emotional impact of literal and sarcastic comments of both valences. The rating scale has been converted so that 0 represents the middle rating of 4, while positive scale numbers represent ratings from 5 to 7, and negative numbers represent ratings from 1 to 3. Error bars represent 95% CI (confidence interval).

In order to test the predictions of the tinge hypothesis, the data were divided into literal and sarcastic comments of both valences, as described above for *Question b*, and then two paired-samples *t* tests were performed between the ratings for sarcastic criticism and literal criticism, and between sarcastic praise and literal praise (see Figure 6). Both *t* tests were significant, showing that sarcastic criticism was perceived as less negative than literal criticism, *t*(35) = 14.7, *p* < .001, and sarcastic praise was perceived as less positive than literal praise, *t*(35) = 12.1, *p* < .001. These results are a replication of those from Experiment 1, and they provide further support for the tinge hypothesis, suggesting that sarcasm also “mutes” the positive or negative nature of sarcastic messages that are more ambiguous.

## GENERAL DISCUSSION

This paper investigated the effect of emoticons and other punctuation marks on the comprehension and emotional impact of sarcastic comments in CMC, where the risk of sarcasm misunderstanding is higher than in FTF communication. In terms of how comments are interpreted, previous research has provided mixed results. For example, whereas Derks et al. (2008) concluded that the wink emoticon makes ambiguous comments seem more sarcastic, Walther and D'Addario (2001) concluded that the wink emoticon does not significantly increase interpretation of ambiguous messages as sarcastic, and Hancock (2004) argued that, instead, ellipsis is the device most associated with sarcasm. In the current paper, we tested a wide variety of textual devices within the same experiments, allowing us to make more specific statements about the role of different devices in computer-mediated communication, and how they interact with other factors such as the valence of the comment itself and the level of contextual support that is provided for a particular interpretation.

Specifically, if the sarcastic comment is placed in a context where the sarcastic meaning of the comment can be easily deduced, adding an emoticon or a punctuation mark to the comment will make no difference to how the receiver will interpret it. Devices such as a wink, tongue face, or ellipsis (but not an exclamation mark) do, however, make unambiguous *literal* comments appear more sarcastic. Alternatively, if a sarcastic comment is written in an ambiguous context (i.e., the receiver cannot tell from the context whether the comment is to be interpreted as literal or sarcastic), accompanying the comment with a wink emoticon (rather than ellipsis or simply a full stop) is a good way of reducing the possibility that it will be misunderstood by the receiver. This suggests that the wink emoticon conveys a sarcastic meaning, which might be useful for clarification purposes when the message is intended as sarcastic but the context is not supportive enough, but less useful when the intended message is literal (in which case using the wink emoticon might confuse the reader and suggest a sarcastic interpretation).

In relation to the perceived emotional impact of sarcastic versus literal language, we have provided consistent evidence that using sarcasm mutes the emotional nature of the message. Specifically, results from both experiments showed that criticism was judged as less negative, and praise as less positive, when uttered sarcastically rather than literally. Therefore, we now have substantial evidence that this muting function is not dependent on the valence of the message or on how strongly the message is supported by context; this is a novel finding and suggests that the muting function of sarcasm applies to both criticism and praise, and irrespective of whether the context is supportive enough of a sarcastic interpretation. In terms of theory, this provides support for the tinge hypothesis (e.g., Dews et al., 1995; Dews & Winner, 1995), suggesting that the use of sarcasm effectively mutes the emotional impact of a message.

These results do, however, contradict previous findings demonstrating an increased emotional impact for sarcastic as opposed to literal comments, including some from our own lab (e.g., Filik et al., 2015). One possible reason for this difference is that Filik et al.’s (2015) study tapped into more immediate responses to sarcastic language than the current study. Specifically, they found that participants’ approach/avoidance responses (that is, the time taken to either pull a lever, indicating an approach response to a positive stimulus, or push a lever, indicating an avoidance response to a negative stimulus) were larger when responding to sarcastic than when responding to literal comments. In contrast, results from the current study and from a number of other rating studies (measuring more considered responses) showed the exact opposite (e.g., Dews et al., 1995; Dews & Winner, 1995; Pexman & Olineck, 2002). Thus, it may be the case that expectations for emotional responses change over time.

Another difference between the current study and other rating studies that instead found enhanced emotional responses for sarcastic as opposed to literal language relates to the harshness of the comments used (see e.g., Bowes & Katz, 2011, who aimed to study extremely harsh sarcastic criticisms). Thus, it is clear that specific factors affecting the emotional impact of sarcasm are something in need of further investigation. However, the current results would suggest that the valence of the comment, or the level of contextual support, are not factors that modulate this process, since a muting effect was found in all instances.

In terms of the influence of emoticons and punctuation marks on perceived emotional impact, results differ depending on the valence of the comment and which device is accompanying it. If a sarcastic comment is written in an ambiguous context, and that comment is intended as criticism, accompanying it with a wink or ellipsis will make the comment seem more negative than a basic full stop. If the sarcastic comment is meant to be a compliment, accompanying it with a wink emoticon (rather than ellipsis or full stop) will increase the positivity of its emotional impact. This would support the findings of Derks et al. (2008), who showed that emoticons enhanced the valence of a message, but would contradict those of Walther and D'Addario (2001) who concluded that they do not.

However, if a sarcastic comment is written in an unambiguous context, irrespective of whether it is meant to be criticism or a compliment, accompanying it with an emoticon (tongue face or wink) or an exclamation mark will make it seem more positive to the recipient than if no emoticon is used. This is partially in line with Carvalho et al. (2009), who include the tongue face emoticon in their category of positive emoticons, and perhaps Garrison et al. (2011), who suggest it indicates playfulness. Interestingly, the presence of an exclamation mark made all comments appear more positive, which would support previous work by Waseleski (2006), which suggested that exclamation marks may function as markers of friendly interaction. The presence of ellipsis did not affect perceived emotional impact.

It is of interest at this point to consider the possible mechanisms via which textual devices may have an influence on the comprehension and emotional impact of sarcastic comments. Since it is the case that materials that are rated as more sarcastic are also perceived as having less of an emotional impact on the recipient (as predicted by the tinge hypothesis), it may be the case that emotional responses were driven by the perception of sarcasm. That is, textual devices influence how sarcastic a comment is perceived to be, which in turn determines the level of emotional impact. Alternatively, it may also be the case that textual devices lead to a particular emotional response, which in turn influences the perceived level of sarcasm. These and other possible mechanisms of action should be the focus of future work.

One further thing to take into consideration for future research is that in Experiment 1, the questions were focused on the comment itself (i.e., *How ironic do you think the final comment is? How do you think the recipient of the comment would feel?*), whereas in Experiment 2, the focus of the question was more on the characters (i.e., *How sarcastic do you think Person A is being? How do you think Person B will respond emotionally?*). It is worth noting that differences in perspective can influence perceived emotional responses to sarcasm (e.g., Bowes & Katz, 2011, although these findings typically relate to victim vs. aggressor perspectives), thus, this is also something that warrants further investigation.

In conclusion, it seems that the comprehension and emotional impact of criticism and praise are influenced by whether the message is intended as literal or sarcastic, and whether it is accompanied by a textual device such as an emoticon. Specifically, the perceived emotional impact of both criticism and praise was muted when the comment was intended sarcastically, rather than literally. In terms of textual devices, results showed that in general, emoticons had a larger influence on both comprehension and emotional impact than punctuation marks.
